# Supplementary figures and images for: Seasonality modulates the predictive skills of diatom based salinity transfer functions
Source: PLoS One. 2018 Nov 20;13(11):e0199343. doi: 10.1371/journal.pone.0199343 (PMC6245675; doi:10.1371/journal.pone.0199343)

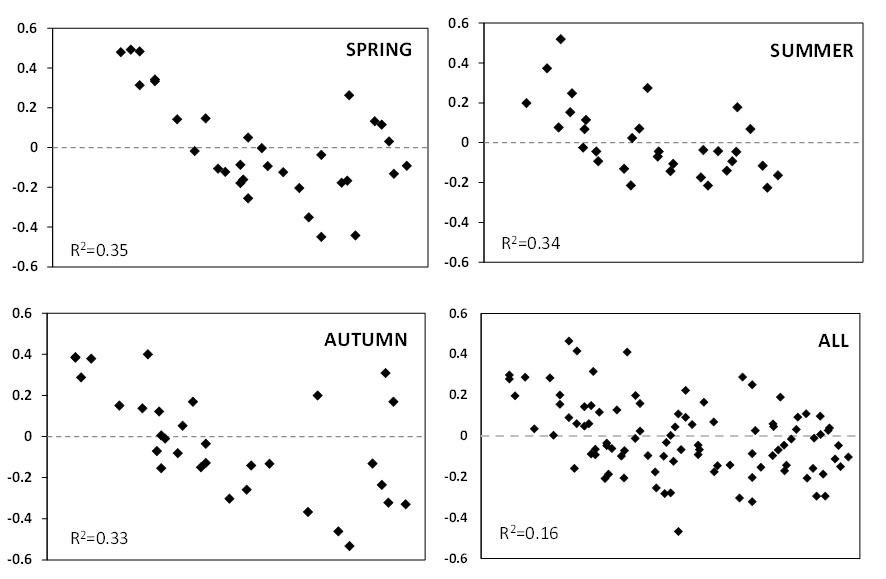

Supplement: S1 Fig — The overestimation of salinity at low end and an underestimation of salinity at the high end of the gradient is especially evident in the spring and autumn models. When all seasons are combined, this edge effects are less prominent and a more accurate performance results. (JPG) [file pone.0199343.s001.jpg]

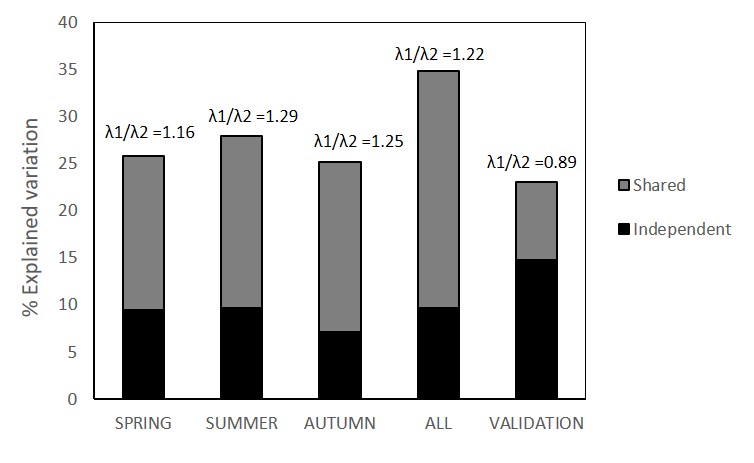

Supplement: S2 Fig — The ratio of λ1/λ2 is also shown for each case. (JPG) [file pone.0199343.s002.jpg]
